# Invariance and plasticity in the *Drosophila melanogaster* metabolomic network in response to temperature

**DOI:** 10.1186/s12918-014-0139-6

**Published:** 2014-12-24

**Authors:** Ramkumar Hariharan, Jessica M Hoffman, Ariel S Thomas, Quinlyn A Soltow, Dean P Jones, Daniel E L Promislow

**Affiliations:** Department of Pathology, University of Washington, Box 357705, Seattle, WA 98195 USA; Laboratory for Integrated Bioinformatics, RIKEN Center for Integrative Medical Sciences, Yokohama, Kanagawa 230-0045 Japan; Department of Genetics, University of Georgia, Athens, GA 30602 USA; Washington University School of Medicine, 660 S. Euclid Avenue, St. Louis, MO 63108 USA; Division of Pulmonary Allergy & Critical Care Medicine, Emory University, Atlanta, GA 30322 USA; Department of Medicine, Clinical Biomarkers Laboratory, Emory University, Atlanta, GA 30322 USA; ClinMet Inc, 3210 Merryfield Row, San Diego, CA 92121 USA; Department of Biology, University of Washington, Seattle, WA 98195 USA

**Keywords:** *Drosophila melanogaster*, Temperature, Metabolomics, Networks, Differential coexpression

## Abstract

**Background:**

Metabolomic responses to extreme thermal stress have recently been investigated in *Drosophila melanogaster*. However, a network level understanding of metabolomic responses to longer and less drastic temperature changes, which more closely reflect variation in natural ambient temperatures experienced during development and adulthood, is currently lacking. Here we use high-resolution, non-targeted metabolomics to dissect metabolomic changes in *D. melanogaster* elicited by moderately cool (18°C) or warm (27°C) developmental and adult temperature exposures.

**Results:**

We find that temperature at which larvae are reared has a dramatic effect on metabolomic network structure measured in adults. Using network analysis, we are able to identify modules that are highly differentially expressed in response to changing developmental temperature, as well as modules whose correlation structure is strongly preserved across temperature.

**Conclusions:**

Our results suggest that the effect of temperature on the metabolome provides an easily studied and powerful model for understanding the forces that influence invariance and plasticity in biological networks.

**Electronic supplementary material:**

The online version of this article (doi:10.1186/s12918-014-0139-6) contains supplementary material, which is available to authorized users.

## Background

Temperature has profound effects on cellular and organismal biochemistry [1], with enzymes functioning best at specific temperatures [2]. The effects of temperature on physiology and fitness have been particularly well-studied in the fruit fly, *Drosophila melanogaster*, which can survive transient exposures to a wide range of temperatures, from −10°C to 40°C [3]. Early studies found a strong negative correlation between survival rate and chronic exposure to high temperatures, while showing that transient exposure to extreme temperatures could increase survival (so-called ‘heat hardening’) [4-6]. Molecular studies have shown that this effect is due, in part, to both heat- and cold-shock inducing the expression of heat shock proteins (HSPs), which help cells to counteract the deleterious effect of thermal shock through multiple mechanisms [7-10].

Over the past several years, researchers have begun to dissect the underlying molecular mechanisms of temperature responses beyond HSPs, with numerous studies in *D. melanogaster* looking at the effect of temperature on the transcriptome, the proteome [11-15] and the metabolome [4,9,16].

The value of studying metabolomic changes in response to temperature shifts in *D. melanogaster* has been demonstrated by numerous studies. For example, it has recently been shown that long-term cold acclimation and heat hardening alters the metabolomic profile of *D. melanogaster* larvae [4,9]. Other studies have sought to identify temperature-associated metabolomic signatures of inbreeding in *D. melanogaster* [4,17]. These metabolomic studies have led to a better understanding of the global effects of the temperature stress response in *D. melanogaster*, moving beyond earlier studies that focused on just a very small number of metabolites in the *D. melanogaster* temperature stress-response [18].

Here we extend these analyses in three ways. First, we take advantage of recent advances in high-resolution mass spectrometry [19-21]. This approach allows us to greatly increase the number of metabolites and metabolic pathways that we can assay for effects of temperature.

Second, we incorporate recent developments in differential network analysis [22,23]. Numerous gene expression studies have shown that in some cases, perturbations can have limited effects on the magnitude of transcripts while causing substantial changes in the correlations between transcripts (e.g., [24]). More recently, researchers have brought this approach to the study of metabolomic networks [25-27]. In light of these results, here we explore not only the structure of the metabolomic network [28], but more specifically, how temperature changes the structure of specific modules within the larger metabolomic network. In particular, we are able to identify modules whose structure is highly constant in response to temperature, as well as modules that change their structure dramatically in response to temperature. Our focus on modules that maintain correlation structure, or alter that structure, in response to temperature, can pave the way to a better understanding of the mechanisms underlying robustness and plasticity of networks in response to environmental changes [29]. To our knowledge, this study is the first to identify temperature-dependent constant and plastic modules in the metabolome, and suggests novel approaches to better understand how poikilothermic organisms might adapt to a changing environment. Moreover, the approaches we use here could lead to important insights into network evolution and the role that network structure plays in the ability of organisms to cope with stressors in general [30].

Finally, by studying the effect of developmental and adult temperature on high-resolution, untargeted metabolomic profiles, we identify novel associations between rearing conditions and metabolites and metabolic pathways.

## Results

Our dataset consisted of data from technical duplicates for 95 samples across four Drosophila Genome Reference Panel (DGRP) genotypes [31]. These included 48 male and 47 female samples, with 24 samples per genotype except for DGRP 25189 which contributed only 23 samples to our analysis. After applying quality control procedures (see Methods), our dataset included 4359 features from a C18 column, and 2961 features from an AE column. We were able to assign putative matches to 1141 metabolites from the C18 column and 926 metabolites from the AE column. We thus had data for a total of 7,320 features with 2027 putatively identified features from both columns, noting that some features overlap between the two columns.

### Pathway enrichment analysis using *mummichog*

For this analysis, we investigated the effects of developmental temperature and adult temperature on male and female flies separately.

#### Developmental temperature effects

**Figure 1:**
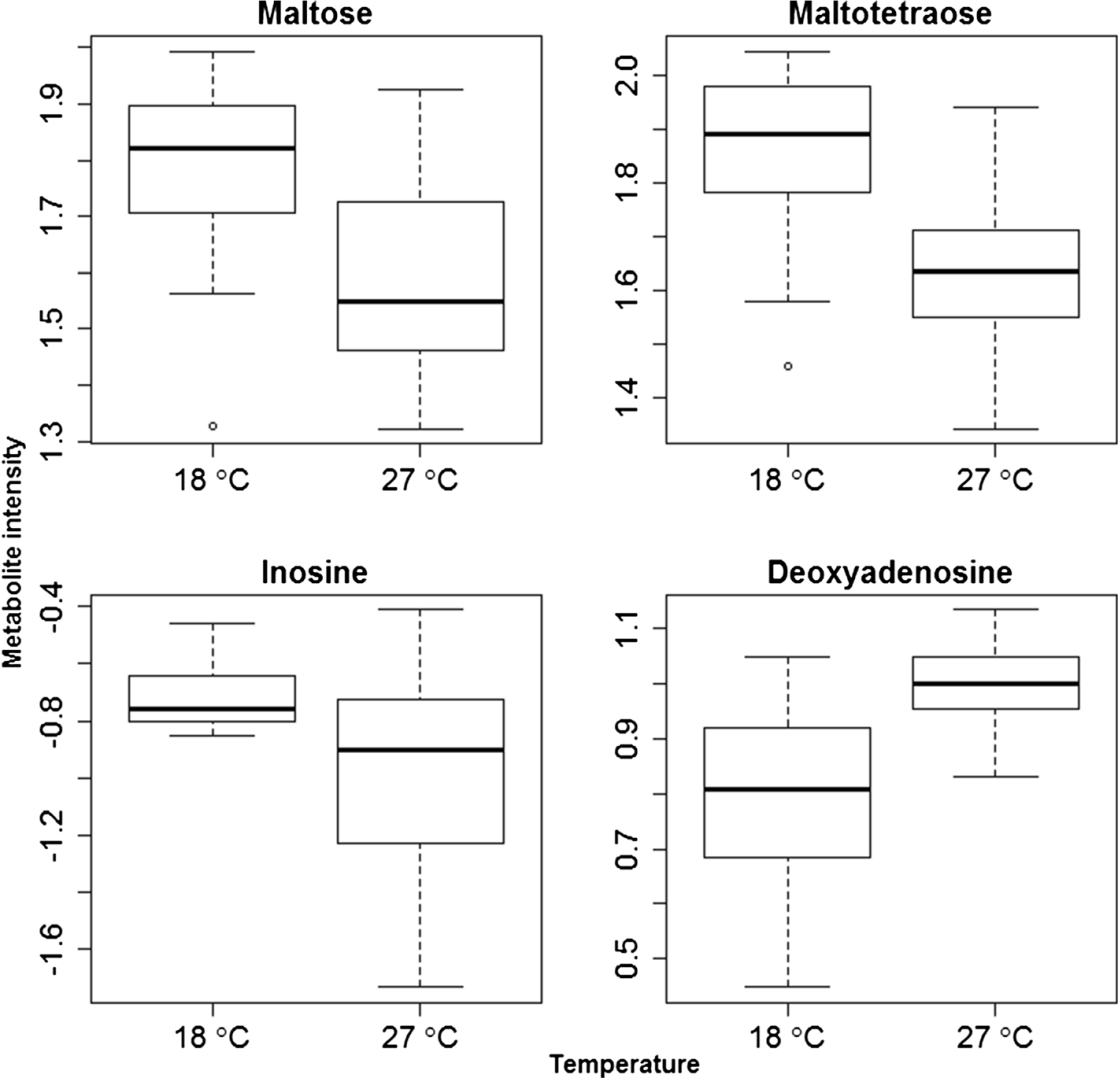
**Effect of temperature on sample metabolite intensities for males (top) and females (bottom).** Metabolite intensities are plotted for each of the four metabolites. All four metabolites had FDR-adjusted p values below 0.01.

For developmental temperature treatment, after controlling for adult temperature and genotype, in adult males we observed 170 and 213 significantly differentially expressed metabolites in the C18 and AE columns, respectively (3.8% and 7.1% of all features). In female samples, we observed 336 and 295 differentially expressed metabolites in the C18 and AE columns, respectively (7.7% and 9.9% of all features). Using *mummichog* [32], we found that metabolites that changed significantly in response to developmental temperature were enriched for six metabolic pathways in male flies, and for seven pathways in female flies (Tables 1 and 2). Two of these pathways, glycogen degradation and trehalose biosynthesis, were found to be affected in both sexes. Specifically, we found higher levels of numerous polysaccharides (maltose, maltotriose, maltotetraose, and trehalose 6-phosphate) in flies raised at lower developmental temperature (Figure 1).

**Table 1:** **Metabolic pathways altered by developmental temperature in males**

**Pathway (a)**	**Overlap size (b)**	**Pathway size (c)**	**Overlap features (id) (d)**
Dopamine degradation	4	10	PAP,3-5-ADP,CPD-782, Adenosyl-homo-Cys
Glycogen degradation I/ Trehalose biosynthesis	3	9	Trehalose-6P, Trehalose
Salvage pathways of adenine, hypoxanthine, and their nucleosides	4	15	AMP, Deoxyadenosine, Xanthine, Adenine
Acyl carrier protein metabolism	2	3	PAP,3-5-ADP
Selenocysteine biosynthesis II (archaea and eukaryotes)	2	4	AMP, Ser

Metabolic pathways (a) identified as enriched in the set of metabolites affected by developmental temperature in adult male flies. The number of metabolites in the input list that overlapped (b) with the reference list of all metabolites after quality control (c), along with the identification of these metabolites (d) is shown.

**Table 2:** **Metabolic pathways altered by developmental temperature in females**

**Pathway (a)**	**Overlap size (b)**	**Pathway size (c)**	**Overlap features (id) (d)**
Glycogen degradation I	8	9	GLC-1-P, Alpha-Glucose, Maltotetraose, GLC, Maltotriose, GLC-6-P, Maltose, Alpha-GLC-6-P
Lactose/Melibiose degradation III	4	4	Lactose, Melibiose, Galactose, GLC
Trehalose biosynthesis I	3	4	Trehalose-6P, Trehalose, Alpha-GLC-6-P
tRNA charging pathway	6	19	Val, Pro, Thr, Phe, Arg, Trp
Salvage pathways of adenine, hypoxanthine, and their nucleosides	7	15	Xanthine, Adenine, Inosine, Deoxyadenosine, Deoxyinosine, AMP, Hypoxanthine
Zymosterol biosynthesis	5	10	Zymosterol,CPD-4702,CPD-4581,CPD-4575,NADP,44-Dimethyl-Choleta-812-24-Trienol
Sphingosine and sphingosine-1-phosphate metabolism	4	8	CPD3DJ-11366, NADP, Sphingosine, Palmitaldehyde

Metabolic pathways (a) identified as enriched in the set of metabolites affected by developmental temperature in adult female flies. The number of metabolites in the input list that overlapped (b) with the reference list of all metabolites after quality control (c), along with the identification of these metabolites (d) is shown.

#### Adult temperature effects

For adult temperature treatment, in males we observed 64 and 67 differentially expressed metabolites in the C18 and AE columns, respectively (1.4% and 2.8% of all features), and in females, 27 and 56 differentially expressed metabolites in the C18 and AE columns, respectively (0.6% and 1.8% of all features). We found that in male flies, adult temperature affected metabolic pathways involved in degradation of purine nucleosides (Table 3). Increased adult temperature in female flies was associated with down-regulation of pathways mediating arginine, 4-hydroxyproline degradation, and NAD biosynthesis.

**Table 3:** **Metabolic pathways altered by adult temperature in males and females**

**Pathway (a)**	**Overlap size (b)**	**Pathway size (c)**	**Overlap features (id) (d)**
*Degradation of purine ribonucleosides	3	10	Adenosine, Guanosine, Adenine
4-hydroxyproline degradation I	3	6	GLT, L-4-hydroxy-proline, NAD
Arginine degradation I (arginase pathway)	3	7	L- glutamate_gamma-semialdehyde, GLT, NAD
NAD biosynthesis from 2-amino-3-carboxymuconate semialdehyde	3	8	Deamido-NAD, GLT, NAD

Metabolic pathways (a) identified as enriched in the set of metabolites affected by adult temperature in adult male (first row, and identified by an asterisk), and female flies. The number of metabolites in the input list that overlapped (b) with the reference list of all metabolites after quality control (c), along with the identification of these metabolites (d) is shown.

#### Warm and cool temperature effects

We further limited the analysis to flies that had experienced the same temperature (either 18°C or 27°C) at both developmental and adult stages. Using the linear model shown in Equation 1, and carried out separate analyses for male and female flies. We identified 96 (C18) and 66 (AE) metabolites in males and 142 (C18) and 78 (AE) metabolites in females whose concentrations were significantly changed by this temperature treatment. *Mummichog* analysis pointed to temperature-dependent changes in three pathways (Tables 4 and 5).

**Table 4:** **Metabolic pathways altered by developmental and adult temperature in males**

**Pathway (a)**	**Overlap size (b)**	**Pathway size (c)**	**Overlap features (id) (d)**
Serotonin and melatonin biosynthesis	4	8	Trp, N-acetyl-serotonin, S-adenosylmethionine, N-acetyl-5-Methoxy-tryptamine

Metabolic pathways (a) identified as enriched in the set of metabolites affected by developmental and adult temperature in male *Drosophila*. For this analysis, we pooled together metabolomics data from flies exposed to cold (18°C) developmental and adult temperatures, and compared them with data from flies exposed to hot (27°C) developmental and adult temperatures. The number of metabolites in the input list that overlapped (b) with the reference list of all metabolites after quality control (c), along with the identification of these metabolites (d) is shown.

**Table 5:** **Metabolic pathways altered by developmental and adult temperature in females**

**Pathway (a)**	**Overlap size (b)**	**Pathway size (c)**	**Overlap features (id) (d)**
Arginine degradation VI (arginase 2 pathway)	3	7	L-glutamate_gamma-semialdehyde, Pro, Arg
Salvage pathways of guanine, xanthine, and their nucleosides	3	9	Deoxyguanosine, GMP, Guanine

Metabolic pathways (a) identified as enriched in the set of metabolites affected by developmental and adult temperature in female *Drosophila*. For this analysis, we pooled together metabolomics data from flies exposed to cold (18°C) developmental and adult temperatures, and compared it with data from flies exposed to hot (27°C) developmental and adult temperatures. The number of metabolites in the input list that overlapped (b) with the reference list of all metabolites after quality control (c), along with the identification of these metabolites (d) is shown.

#### Genotype effects

We were also interested in identifying metabolites whose intensities were affected significantly by genotype, i.e., metabolites whose levels differ among genotypes. In males, we identified 354 (C18) and 297 (AE) metabolites, while in females we detected 355 (C18) and 364 (AE) metabolites that exhibited significant differential abundance. Significantly enriched metabolic pathways in these sets of metabolites were identified using *mummichog* (Additional file 1). Seven and five metabolomic pathways were detected as being significantly affected by genotype in female and male flies respectively.

By contrast, we found very few metabolites whose temperature response was genotype-dependent. Specifically, we detected less than six metabolites each from the interaction analysis between different factors (genotype, developmental temperature, and adult temperature).

### DiffCoEx analysis

**Figure 2:**
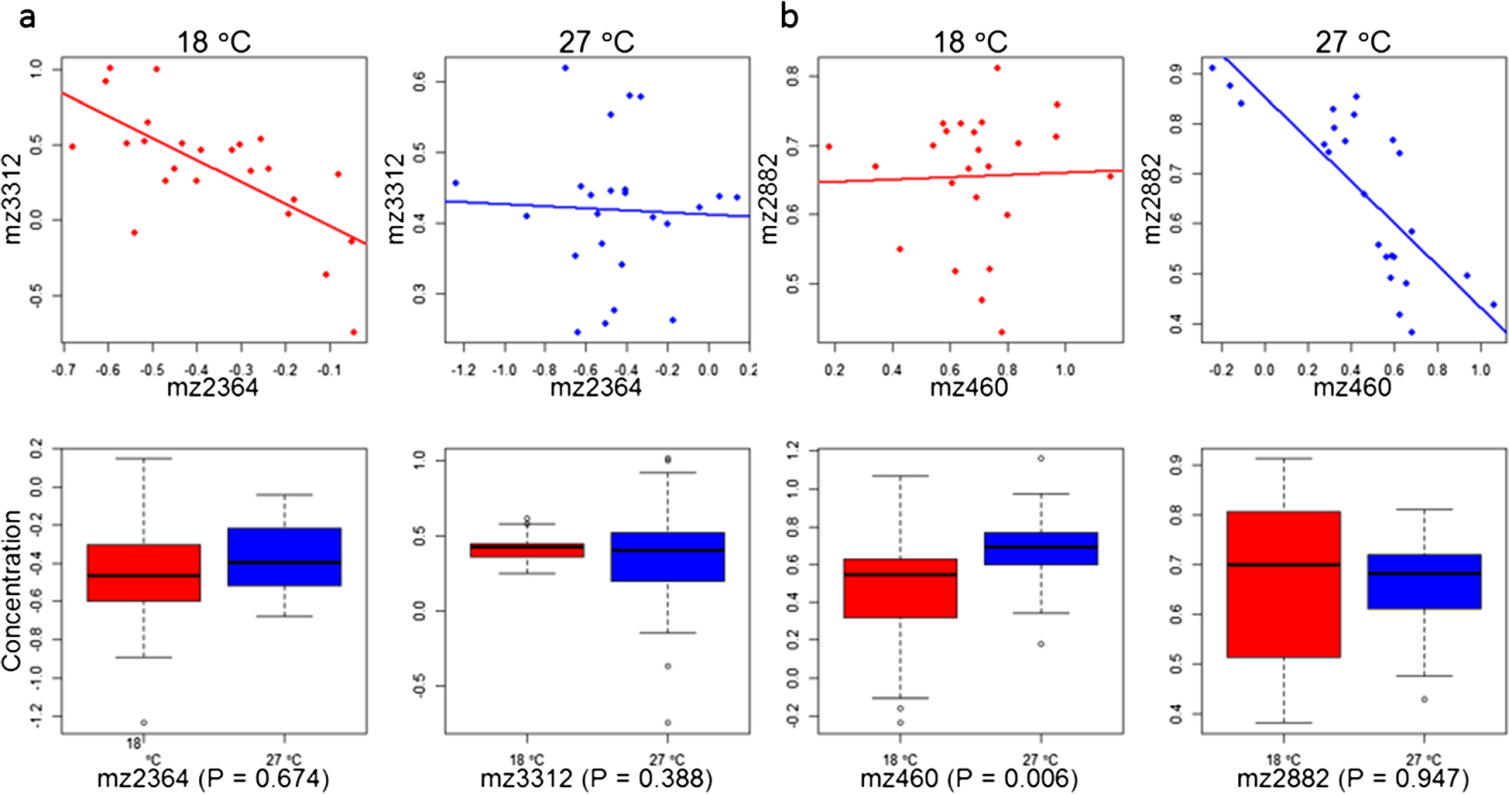
**Correlation and main effects for a pair of metabolites from male (a) and female (b) fly metabolomics data.** *Top panel*: Correlation between two metabolites at 18°C (left) and 27°C (right). Lines are least-squares regression. *Bottom panel*: Effect of temperature on each of these two metabolites.

**Figure 3:**
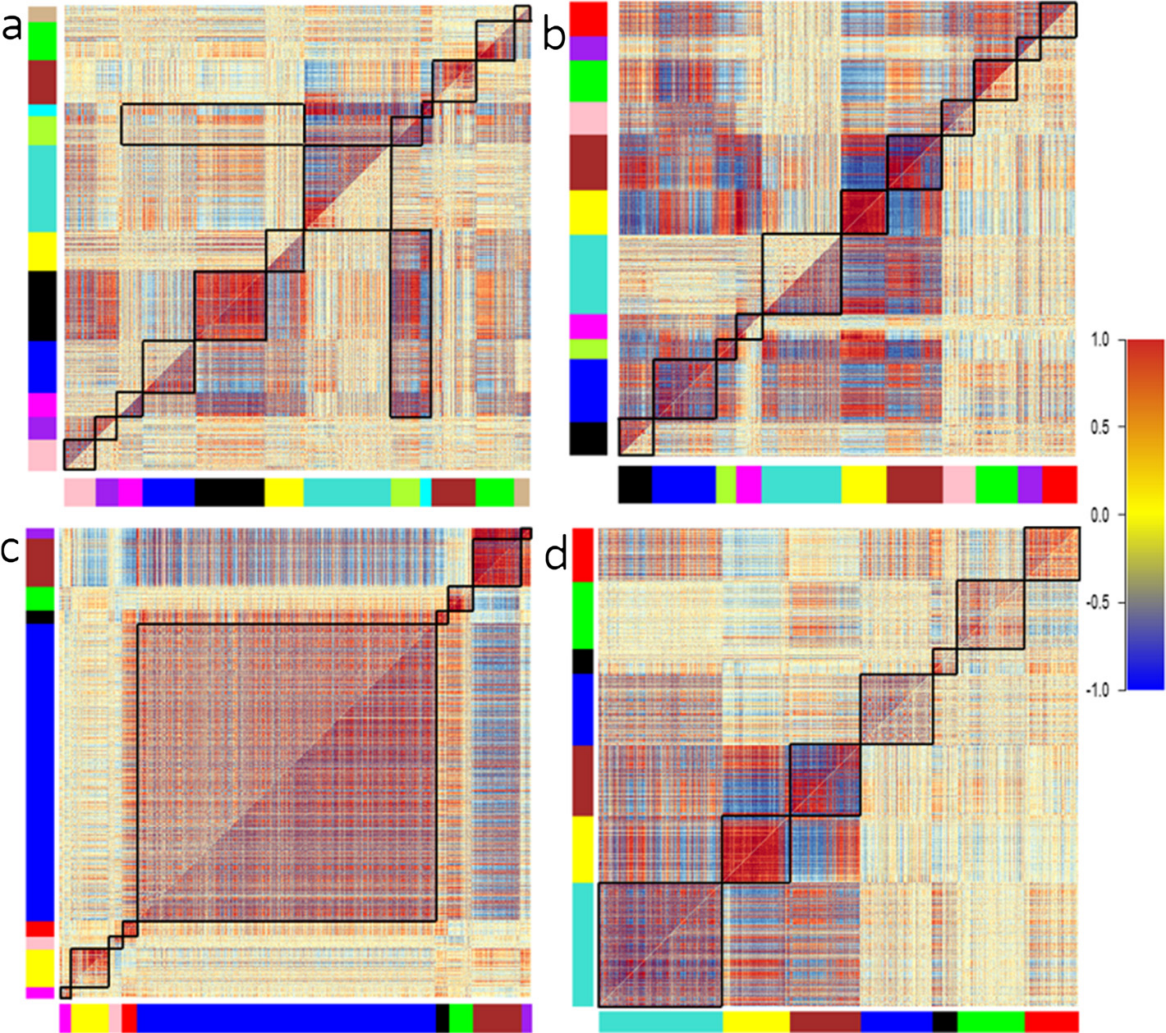
**Differentially co-expressed (a and b) and preserved (c and d) metabolite modules of metabolites in males (a and c) and females (b and d) in response to developmental temperature.** The heat maps consist of an *N* x *N* grid of *N* metabolites, and each pixel represents the correlation coefficient across samples between any two metabolites (red is positive, blue is negative). The metabolites are ordered such that groups of highly correlated metabolites (modules) are clustered together. The top left represents correlations between pairs of metabolites at developmental temperature of 18°C., and the bottom right at 27°C. To identify modules that change or are preserved significantly between the two conditions, we used the R package DiffCoEx (see Methods). Modules of metabolites are depicted as black squares along the central diagonal, and also as colored boxes on the bottom and on the left. A correlation color scale is shown on the right, with red corresponding to *r* = 1, and blue to *r* = −1.

Among metabolites whose concentrations were not affected by temperature, we found many pairs that showed changes in correlation coefficients between temperature treatments (Figure 2). To systematically investigate the correlation effects, we carried out a metabolome-wide analysis of differentially co-expressed metabolites. Using the R package DiffCoEx [22] for differential network analysis, in male flies, comparing the two developmental temperatures, we detected 12 differentially co-expressed modules from a network of 1363 metabolites (Figure 3a). For the female fly metabolome, we identified 11 such network modules from a total of 814 metabolites (Figure 3b). After permutation tests for significant differences (Methods) in correlation structure between temperature, ten of the 12 male modules and seven of the 11 female modules showed statistically significant change in response to developmental temperature (Additional file 2).

**Figure 4:**
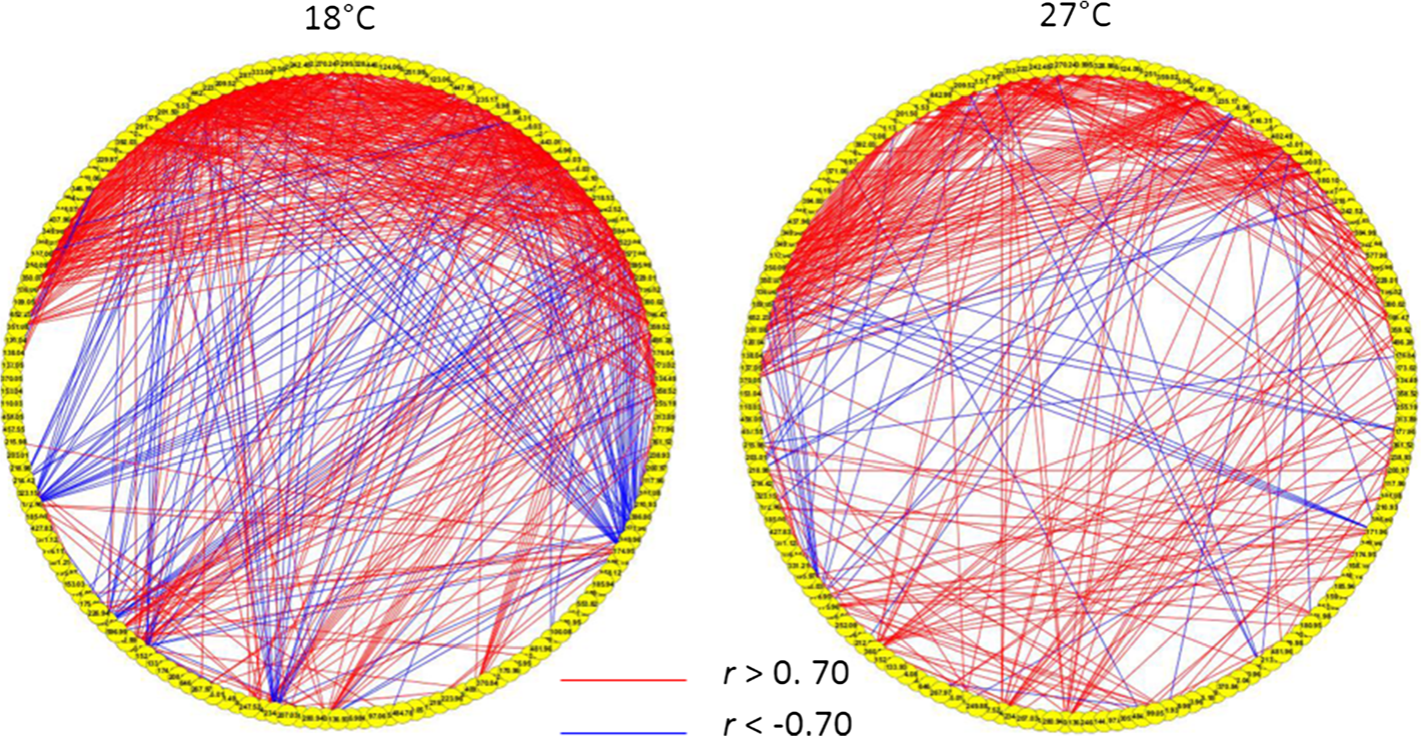
**Preserved module in female fly metabolome.** Architecture of the brown module, a highly preserved module in the female fly metabolome between 18°C (left) and 27°C (right). Nodes represent metabolites and edges represent correlations between the node pairs. Only correlation values of *r* ≥ 0.7 (red) or *r* ≤ −0.7 (blue) are shown. The location and relative order of each node in the module are the same across the two temperature conditions.

Using a slightly modified approach with DiffCoEx, we identified nine male modules and seven female modules whose structure was markedly *preserved* (‘Similarity matrices’) between the developmental temperature conditions (Figures 3c, d and 4 and Additional file 3).

For illustrative purposes, we visualized representative DiffCoEx and Similarity matrix modules in Cytoscape [33], choosing the smallest of the detected modules for each sex. Comparison of the magenta module (Figure 3a and Additional file 4) from the male fly metabolome reveals that this module becomes much more tightly correlated when developmental temperature changes from 18°C to 27°C. In contrast, a visual comparison of the red module (Figure 3b and Additional file 5) for female samples suggests that this module loses a large proportion of its correlations when developmental temperature becomes warmer. Cytoscape based network visualizations for the most striking of the Similarity matrix modules for the male and female metabolomes are shown in Figure 4.

For each DiffCoEx and Similarity matrix modules, we used *mummichog* to test for metabolic pathway enrichment. We found significant enrichment in six out of 12 DiffCoEx modules in the male fly data, and in three out of 11 DiffCoEx modules in the female fly data (Additional file 6 and Additional file 7). Several novel pathways not detected by differential expression analysis were detected as significant in the network analysis, including glycolysis, lipoate biosynthesis and N-acetyl-glucosamine degradation. We did not detect any enriched metabolic pathways among the preserved models.

## Discussion

In this study, we have carried out two levels of analysis. The first level of analysis allowed us to identify main effects of temperature on metabolite levels. The second level of analysis, focused on the metabolomic network, enabled us to ask questions about network invariance and plasticity.

We investigated metabolomic thermal responses to moderate temperatures (27°C and 18°C). In contrast to earlier studies that have focused on the effects of stressful or extreme temperatures on the fly metabolome, here we focus on longer term exposure to temperatures that are considered to be relatively benign for this organism [34]. Thermal responses have been extensively investigated in *D. melanogaster*, mostly looking at the effects of stressful temperatures during both development and adulthood on fly physiology [7,35-37]. The metabolomic effects of heat-shock, rapid cold hardening, long-term cold acclimation, and of benign and stressful temperatures on inbreeding, have all been previously studied in *D. melanogaster* [4,9,17]. In addition to these metabolomic studies, molecular mechanisms of many thermal responses in the fly have been investigated at the level of the transcriptome, and more recently, the proteome [11-15,38]. One benefit of focusing on less extreme temperatures is that we can gain insight into ways that the metabolome responds to natural variation in temperature specifically, rather than response to stress.

### Main effects of temperature on the metabolome

Several metabolic pathways are known to be affected by temperature in *D. melanogaster*, and our analysis looking at main effects of temperature recapitulated many of these earlier findings while extending them in new directions.

From previous studies we know that brief exposures to stressful temperatures, including cold stress, can cause accumulations of polysaccharides in *D. melanogaster* [9,17]. Mechanisms by which maltose and trehalose protect cells during stressful conditions have been described previously [17]. Stressful temperatures can also cause accumulations of proline in flies [9].

Cold acclimation in *D. melanogaster* is also known to be associated with significant metabolic changes involving sugars (sucrose, fructose, and trehalose), polyamines and a few metabolic intermediates [16]. While that study used targeted metabolomics, results from our untargeted metabolomics approach also point to altered trehalose biosynthesis in both male and female flies reared at a lower developmental temperature. Our results suggests that these pathways play a role not only in the response to acute, potentially lethal stressors, but also to the range of temperatures a fly is likely to encounter from day to day.

A recent study implicated dopamine in modulating behavioral response to temperature changes in *D. melanogaster* [39]. We also observed changes in dopamine metabolism with thermal responses in *D. melanogaster*. The agreement between our study and the earlier study supports a role for dopamine in thermal responses in the fly, which warrants deeper investigation in future studies. Specifically, future work should examine the effect of up- or down-regulation of dopamine [40] on temperature-dependent fitness.

Earlier studies have observed that cold acclimation can alter the glycerophospholipid composition of biomembranes [9,38]. In line with these earlier findings, our results suggest that membrane sphingolipid metabolism might be important in how flies respond to changes in developmental temperature. Taken together with earlier studies, it seems likely that temperature has a major effect on cell membrane biochemistry. Interestingly, the role of sphingosine might go beyond membrane structure, as it is also involved in diverse signaling processes [41,42].

We also observed a significant effect of developmental temperature on zymosterol metabolism. While zymosterol is a key intermediate in cholesterol [41], it is likely that zymosterol in flies was bioaccumulated from yeast in fly media [43]. This suggests that in *D. melanogaster*, developmental temperature modulates bioaccumulation of zymosterol from fly media, a hypothesis one could test by adding zymosterol to yeast-free holidic fly medium [44].

In contrast to developmental temperature, the effect of adult temperature on the metabolome was more modest (Table 3). This might be because adult flies were not exposed to the different temperatures for the same duration nor over different developmental stages, and developing fly larvae might be biochemically more malleable than adults. In our comparison of lifetime temperature exposure (combining samples with the same developmental and adult temperature), we observed changes in several metabolic pathways (Tables 4 and 5), at least one of which is of potentially interesting biological relevance. Spermine is a polyamine formed from spermidine and has been shown previously to mediate stress resistance in *Drosophila* [45].

These findings come with an important caveat. As with all metazoan species, the fruit fly metabolome has yet to be fully curated. We do not know how many metabolites are in the fly, and of those we have measured, most have unknown identities. *Mummichog* uses a metabolic pathways and network-based approach to find enriched pathways and annotate metabolites [32]. For the majority of metabolites in the enriched pathways calculated by *mummichog*, we were able to find independent confirmation of their identities by mining the METLIN database using their m/z values (Additional file 8) [46]. While validation of all putative matches is an important goal, it is beyond the scope of this current study, but highlights the need for a curated fly metabolome.

The fact that we confirm previous findings allows us to place greater confidence in our non-targeted, high-throughput analysis, as well as in the novel biological insights gained from our study. We report developmental temperature effects on several pathways not previously associated with temperature response. These include salvage pathways of adenine, hypoxanthine and their nucleosides, and acyl carrier protein metabolism. These findings suggest novel hypotheses for temperature adaptation that one could test through knock-down of fly genes coding for enzymes used in these pathways.

### Effect of genotype

We were primarily interested in investigating the effects of developmental and adult temperature in the fly metabolome. While we found strong effects of temperature on the metabolome, genotype proved to affect an even greater number of metabolites than temperature. Interestingly, most of the pathways affected by genotype do not overlap with those modified by temperature. To our surprise, very few metabolites showed significant genotype-by-temperature interactions. Thus, our data suggest that the effect of temperature on the metabolome is consistent across genotypes. The lack of genotype-by-temperature effects might be due, in part, to the fact that we only included four genotypes in this study. Studies on a broader array of genotypes are needed to rule out the effect of genotype on temperature effects both on absolute metabolite levels and metabolite network structure.

### Effect of temperature on network structure

To go beyond the identification of main effects, we performed a second level of analysis, examining the effect of temperature on the structure of metabolite co-expression networks and carrying out detailed analysis on such networks in response to temperature.

Differential co-expression analysis of metabolomic data is relatively novel [28,47], and until now has not been used to identify pathways associated with environmental perturbations. Our analysis has allowed us to develop novel hypotheses for metabolite modules that respond to temperature. Our network analysis identified both highly invariant as well as highly plastic metabolite modules. Our discovery of metabolite modules that were significantly preserved across the two temperature conditions suggests that there are not just individual metabolites, but large modules whose coordinate function is invariant in the face of at least mild temperature changes.

Importantly, we do not know whether the invariant correlation structure among modules is an adaptation that facilitates homeostasis, if the correlations are neutral, or if, in fact, the failure of those correlations to change in the face of different temperatures is actually maladaptive [48]. To distinguish between these hypotheses, one would need to compare temperature-dependent fitness in groups in which the correlation structure was broken, either through dietary or genetic manipulation of two or more metabolites simultaneously, or by comparing genotypes (such as the DGRP [28]) in which levels of two or more metabolites deviate to a greater or lesser extent from that predicted from these correlations.

While the preserved modules found in this study cannot directly answer questions about network robustness, they can serve as a foundation for carrying out further experimental and computational studies to glean deeper insights into network robustness in response to temperature changes.

## Conclusions

In summary, our results demonstrate that high resolution metabolomic profiles coupled with differential co-expression network analysis provides a powerful tool to understand the molecular response to shifts in temperature. Transcriptomic studies have used different methods to detect and quantify differentially co-expressed genes in a wide variety of settings, from cancer tissues [24,49], to Alzheimer’s disease [50], to cardiovascular disease [51]. Here we show that network correlation analysis developed for gene expression data [23] can successfully be extended to analyze metabolomic data to address important biological questions.

Others have already noted that the metabolome offers a powerful intermediate step to link genetic and environmental variation to downstream phenotypes [52]. This study illustrates how network approaches can further increase that explanatory power, suggesting novel hypotheses to be tested in future. Of course, we still have far to go—only a fraction of all metabolites are known, even in humans. As metabolomics catches up with genomic and proteomic technology, network analysis is likely to tell us much about organismal function.

## Methods

### Fly stocks

All experiments were carried out using four genetically distinct and well characterized inbred strains (FlyBase 25180, 25184, 25189, and 25198) that are part of the *Drosophila* Genetic Reference Panel (DGRP) [31]. Fly stocks were maintained in glass vials at 24°C on a 12/12 light–dark cycle at approximately 50% humidity. Flies were cultured on standard yeast-molasses-agar-cornmeal medium with propionic acid added as an anti-fungal agent.

### Temperature treatments

For each strain, eight fresh vials were created, with ten males and ten females in each. After 48 hours of copulation and egg laying at 24°C, adults were discarded. The vials containing eggs were placed at either 18°C or 27°C until eclosion. Virgin collections for adult flies at developmental temperatures 27°C and 18°C were initiated at 10 and 19 days, respectively, following incubation at that temperature. Virgins were collected from each developmental temperature, and then distributed equally between the 18°C and 27°C incubators. This resulted in four temperature treatment groups: 18°C → 18°C, 18°C → 27°C, 27°C → 18°C, 27°C → 27°C. Adult flies were kept at this temperature for four to five days before being frozen in liquid nitrogen, and stored at −80°C.

### Metabolomic analysis

Sample preparation and analysis were carried out as described previously [53]. Briefly, acetonitrile extracts from frozen samples of three adult whole body *D. melanogaster* were analyzed in a dual column chromatography-mass spectrometry (LC-MS) platform. In this DC/LC-MS platform, the C18 (or ‘reverse phase chromatography’) column retains and separates chemicals with partial hydrophobic character, and the Anion Exchange (AE) column retains and separates negatively charged analytes. Fractionated samples, after electrospray ionization, were detected using a Fourier-transform mass spectrometer. Data were extracted as non-annotated mass/charge (*m/z*) features, column retention time, and ion intensity. Fly standards were also run alongside investigational samples. Specifically, we made a pooled reference sample and stored a large number of aliquots at −80°C to allow comparison of analytic behavior over long periods of time. The pooled reference samples were for the purposes of quality control (i.e., to ensure relative consistency among identical samples within days) and for quality assurance (i.e., to ensure consistent results between days). They did not contribute data to downstream statistical analysis.

### Data analysis

#### Quality control

Since the column chemistries are different, we analyzed data from the C18 and AE columns, separately. First, we applied various quality control procedures to the data, following [49]. We included only those metabolites with a signal-to-noise ratio of at least 14. We then log-transformed the data and removed any analytes missing from more than 5% of male or more than 5% of female samples. After this, between 0.28% (AE) and 0.31% (C18) of all cells consisted of missing values (i.e., missing fewer than 1 in 300 m/z values in each case). To impute these missing values, we used the ‘EMimpute array’ method in the LSimpute algorithm [54].

#### Statistical analysis

Statistical analyses were carried out using the statistical package R (R Development Core team 2008) and Microsoft Excel 2013.

After technical replicates were averaged and collapsed, we had metabolite measurements from 95 samples. Samples from male and female flies were analyzed separately. Apart from sex, there were three known, independent variables in our study, including developmental temperature (*L*), adult temperature (*A*), and genotype (*G*). We tested for the effects of these three variables using a linear model treating all three factors as fixed effects, and including all pairwise interactions:

1 $$ {y}_i=\mu +L+A+G+L\mathrm{x}A+L\mathrm{x}G+A\mathrm{x}G+\upepsilon $$

where *y*_*i*_ is the intensity of metabolite *i, μ* is the mean, and *∊* is the error.

To identify differentially expressed metabolites for each factor in equation 1, we used a conservative false discovery rate (FDR) threshold of 0.01 [55]. To investigate the effect of genotype on temperature treatment and its interactions, we carried out a likelihood ratio test [53].

#### Metabolic pathway enrichment and metabolite annotation

We sought to identify metabolic pathways that were enriched within the sets of differentially expressed metabolites. To do this, we used the program *mummichog*, which provides putative annotation for metabolites based on mass-charge ratios and carries out statistical tests for enrichment [32]. *Mummichog* provides adjusted *P-*values, correcting for the fact that different *m*/*z* features can map to a single metabolite and single *m/z* values can map to different metabolites [32]. *Mummichog* uses BioCyc (biocyc.org) as a source for metabolite ontologies where some of the fly metabolic pathways in BioCyc are based on experimental evidence while others are inferred. We chose an adjusted P-value cutoff of 0.01 to identify enriched metabolic pathways from *Mummichog* output.

#### DiffCoEx analysis

We used the DiffCoEx algorithm to identify sets of metabolites or modules whose correlation structure changed significantly across experimental conditions [22]. DiffCoEx runs on the Weighted Gene Correlation Network Analysis (WGCNA) platform, which is an R-based software package designed for network analysis [23]. To calculate the significance of the co-expression differences within and between modules, we used permutation testing. Each dataset was permuted 1000 times for each temperature condition. The dispersion value (*d*) for each module and for each pair of modules were calculated. P-values were calculated as the proportion of permuted dispersion values greater than or equal to the original *d*_*.*_ As before, we used *mummichog* to identify metabolic pathways that were enriched within specific modules. We also used a slight modification of DiffCoEx to detect significantly *preserved* modules in both male and female fly metabolomes. To do this, we modified the DiffCoEx algorithm to calculate a Similarity Matrix ***S*** instead of a dissimilarity matrix. Modules whose correlation structure changes between conditions were defined by hierarchical clustering on a difference matrix, ***D***, where ***D*** = |(***A*** − ***A***^'^)/2|^β/2^, where ***A*** and ***A***’ are the adjacency matrixes under two conditions and *β* is the soft-thresholding parameter (from WGCNA [23]), set to *β* = 5 in all of our analyses. *β* is a positive integer and serves to transform the correlations so that weights of large correlation differences are given more emphasis compared to smaller differences which are less meaningful. Higher values of *β* increase statistical stringency by placing lower emphasis on smaller correlation differences. To identify modules with constant structure across treatments, we calculated a similarity matrix, ***S*** = (1 − ***D***) * |***A***| * |***A***^'^|. By this definition, pairs of metabolites whose correlations are similar and high (i.e., *r*_*ij,18* ≈_*r*_*ij,27*_ → 1, where *r*_*ij,t*_ is the correlation coefficient between two metabolites *i* and *j* at temperature *t*), are weighted more heavily than pairs of metabolites whose correlations are similar but non-significant (i.e., *r*_*ij,18* ≈_*r*_*ij,27*_ → 0). At the limit where *r*_*ij,18* =_*r*_*ij,27*_, ***S***_***ij***_ = *r*^2^.

To visualize representative modules, we used Cytoscape [33]. Metabolites were defined as nodes, and the correlation between any two metabolites was treated as a weighted edge in Cytoscape.

For a detailed description of DiffCoEx parameters used in this study, see Additional file 9.

## Additional files

Additional file 1:
**List of metabolites in each of four** ***D. melanogaster*** **genotypes that were significantly affected by developmental temperature.**
Additional file 2:
**Permutation testing to assess significance of within and module-to-module co-expression changes associated with developmental temperature treatment in male (top) and female (bottom) metabolomes.** Dispersion values are calculated for each module, and also across all module pairs. This figure allows us to assign *P*-values for within-module, and module-to-module changes in co-expression. Dark grey color represents statistically significant P-values, whereas lighter shades represent numerically higher values for *P*. Dividing values given in the shaded blocks by 1000 directly gives the *P* value for that comparison based on 1000 permutations. For details of the procedure, see [22]. Additional file 3:
**Preserved module (red) in male fly metabolome.** Architecture of a significantly preserved module in the male fly metabolome across two conditions of developmental temperature, 18°C (left) and 27°C (right). Edges are colored as in Figure 3. Both location and relative order of each node in the module are the same across the two temperature conditions. Additional file 4:
**Changes in co-expression between metabolite pairs in specific network modules in male fly metabolome.** Top panel: Within module changes in co-expression in the magenta module across two conditions of larval temperature, 18°C (left) and 27°C (right). Edges are colored as in Figure 3. The location and relative order of each node in the module are the same across the two temperature conditions. Bottom panel: Changes in correlation among a subset of annotated metabolites from the magenta module. Mass to charge values (mz values) are shown inside the nodes. Annotated mz’s are 96.5143 (oxalosuccinate), 137.0957 (estradiol-17-beta), 113.1067 (spermine), 447.3451 (3-dehydroteasterone), 111.0799 (histamine), 281.0539 (thymidine), and 119.0346 (D, L- malic semialdehyde). Additional file 5:
**Changes in co-expression between metabolite pairs in specific network modules in female fly metabolome.** Top panel: Within module changes in co-expression in the red module across two conditions of larval temperature, 18°C (left) and 27°C (right). Edges are colored as in Figure 3. Both location, and relative order of each node in the module are the same across the two temperature conditions. Bottom panel: Changes in correlation among a subset of annotated metabolites from the magenta module. Mass to charge values (mz values) are shown inside the nodes. Annotated mz’s are 423.0885 (adenosyl- homocysteine), 365.1036 (lactose/maltose/melibiose/sucrose), 527.1572 (maltotriose), 381.0771 (lactose/maltose/melibiose/sucrose), 181.0713 (glucose/galactose), 505.1738 (maltotriose), and 667.2271 (maltotetraose). Additional file 6:
**Metabolic pathways (b), enriched in differentially co-expressed modules (a) in male** ***Drosophila*** **.** These modules were identified by the DiffCoEx algorithm [22], as significantly altered in response to developmental temperature exposures. The number of metabolites in the input list that overlapped (b) with the reference list of all metabolites after quality control (c), along with the identification of these metabolites (d) is shown. Additional file 7:
**Metabolic pathways (b), enriched in differentially co-expressed modules (a) in female Drosophila.** These modules were identified by the DiffCoEx algorithm [22], as significantly altered in response to developmental temperature exposures. The number of metabolites in the input list that overlapped (b) with the reference list of all metabolites after quality control (c), along with the id of these metabolites (d) is shown. Additional file 8:
**Mass-to-charge ratios of metabolites in the enriched pathways detected by** ***mummichog*** **and status of their identity confirmation using the METLIN database.**
Additional file 9:
**Details of the DIFFCOEX algorithm used to identify both differentially co-expressed and significantly preserved modules in this study.**
