# Supplementary material for: Invariance and plasticity in the Drosophila melanogaster metabolomic network in response to temperature
Source: BMC Syst Biol. 2014 Dec 24;8:139. doi: 10.1186/s12918-014-0139-6 (PMC4302152; doi:10.1186/s12918-014-0139-6)
Supplement: Additional file 1: — List of metabolites in each of four D. melanogaster genotypes that were significantly affected by developmental temperature. [file 12918_2014_139_MOESM1_ESM.docx]

List of all four line-specific metabolites in *D. melanogaster* that were significantly affected by developmental temperature.

| **DGRP 25180** | **DGRP 25185** | **DGRP 25189** | **DGRP 25198** |
| --- | --- | --- | --- |
| 503.3767026 | 499.779167 | 474.2043372 | 333.1139667 |
| 384.2729563 | 271.0763204 | 457.1720438 | 112.0174251 |
| 488.1712617 | 483.3774037 | 600.4647543 | 189.0733938 |
| 218.1362251 | 499.2793877 | 89.46491387 | 295.1123369 |
| 464.2744687 | 395.3096745 | 137.9636976 | 600.4647543 |
| 504.7536525 | 524.3583035 | 112.9845461 | 213.1455761 |
| 491.1846213 | 483.314525 | 95.9728695 | 205.1244043 |
| 547.1997898 | 209.9831397 | 112.0174251 | 136.0388026 |
| 384.7457454 | 359.102031 | 124.9557775 | 417.2360442 |
| 358.2541351 | 259.6306245 |  | 176.1175802 |
|  | 247.2412074 |  | 200.0396782 |
|  | 281.2659999 |  | 691.5075722 |
|  | 481.3103393 |  | 433.1728655 |
|  | 124.9992975 |  | 204.1224623 |
|  | 463.2627454 |  | 278.0854794 |
|  | 260.1324395 |  | 89.03814183 |
|  | 85.02785099 |  |  |

**Additional file 2**. Mass-charge ratios of metabolites detected as significantly differentially expressed between flies exposed to different developmental temperatures. We were unable to detect any significantly enriched metabolic pathways in these sets of metabolites using *mummichog* (32). We used an FDR threshold of 0.01 to identify these metabolites.
