# Supplementary figures and images for: Invariance and plasticity in the Drosophila melanogaster metabolomic network in response to temperature
Source: BMC Syst Biol. 2014 Dec 24;8:139. doi: 10.1186/s12918-014-0139-6 (PMC4302152; doi:10.1186/s12918-014-0139-6)

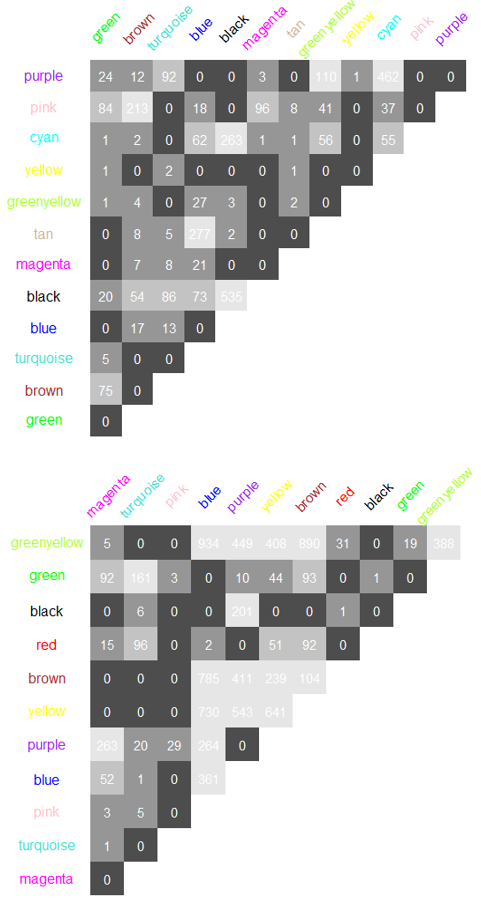

Supplement: Additional file 2: — Permutation testing to assess significance of within and module-to-module co-expression changes associated with developmental temperature treatment in male (top) and female (bottom) metabolomes. Dispersion values are calculated for each module, and also across all module pairs. This figure allows us to assign P-values for within-module, and module-to-module changes in co-expression. Dark grey color represents statistically significant P-values, whereas lighter shades represent numerically higher values for P. Dividing values given in the shaded blocks by 1000 directly gives the P value for that comparison based on 1000 permutations. For details of the procedure, see [22]. [file 12918_2014_139_MOESM2_ESM.tiff]

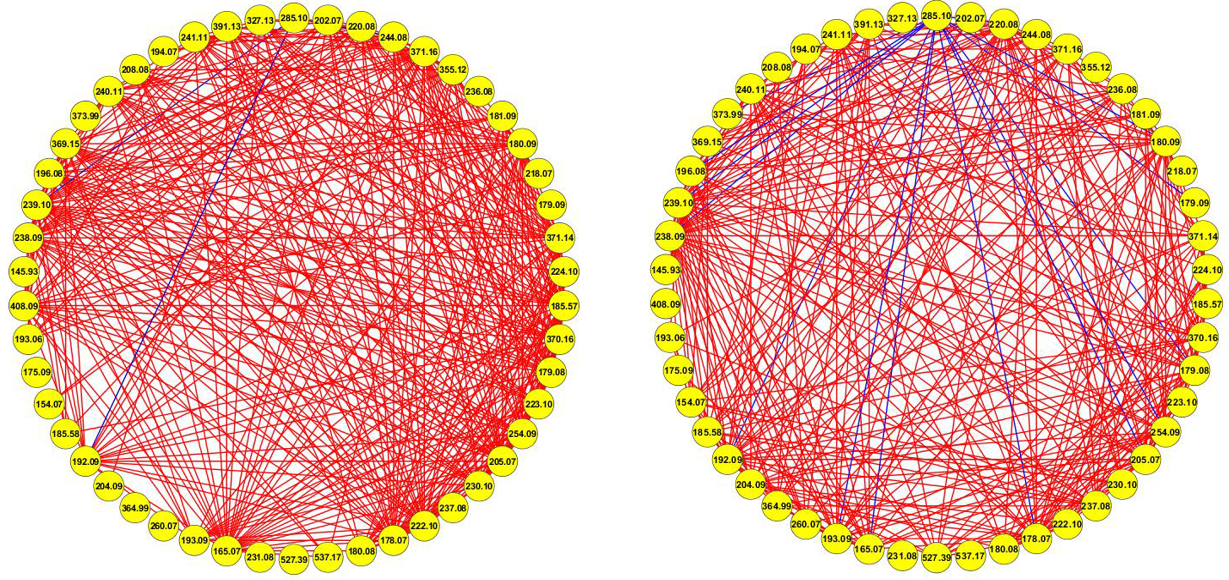

Supplement: Additional file 3: — Preserved module (red) in male fly metabolome. Architecture of a significantly preserved module in the male fly metabolome across two conditions of developmental temperature, 18°C (left) and 27°C (right). Edges are colored as in Figure 3. Both location and relative order of each node in the module are the same across the two temperature conditions. [file 12918_2014_139_MOESM3_ESM.tiff]

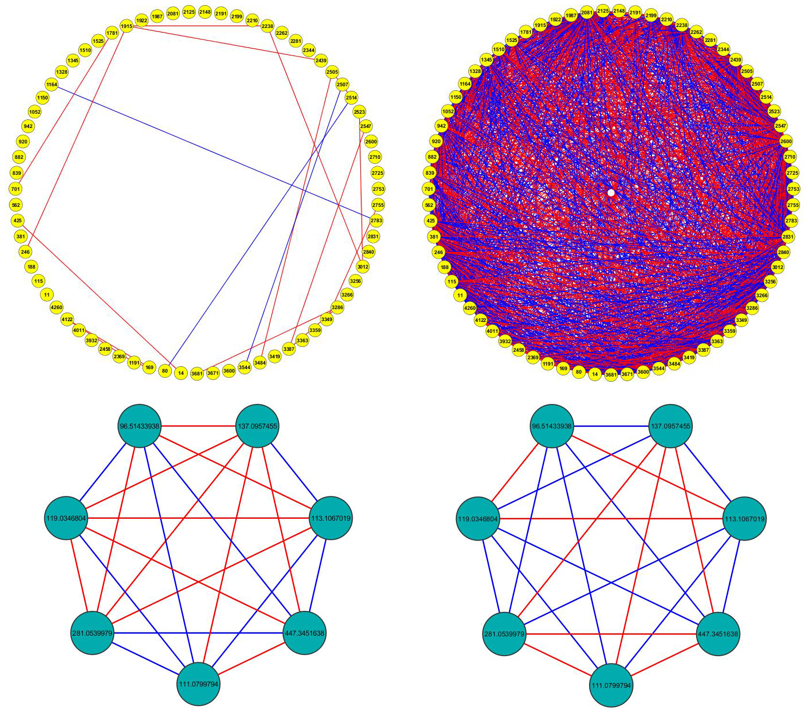

Supplement: Additional file 4: — Changes in co-expression between metabolite pairs in specific network modules in male fly metabolome. Top panel: Within module changes in co-expression in the magenta module across two conditions of larval temperature, 18°C (left) and 27°C (right). Edges are colored as in Figure 3. The location and relative order of each node in the module are the same across the two temperature conditions. Bottom panel: Changes in correlation among a subset of annotated metabolites from the magenta module. Mass to charge values (mz values) are shown inside the nodes. Annotated mz’s are 96.5143 (oxalosuccinate), 137.0957 (estradiol-17-beta), 113.1067 (spermine), 447.3451 (3-dehydroteasterone), 111.0799 (histamine), 281.0539 (thymidine), and 119.0346 (D, L- malic semialdehyde). [file 12918_2014_139_MOESM4_ESM.tiff]

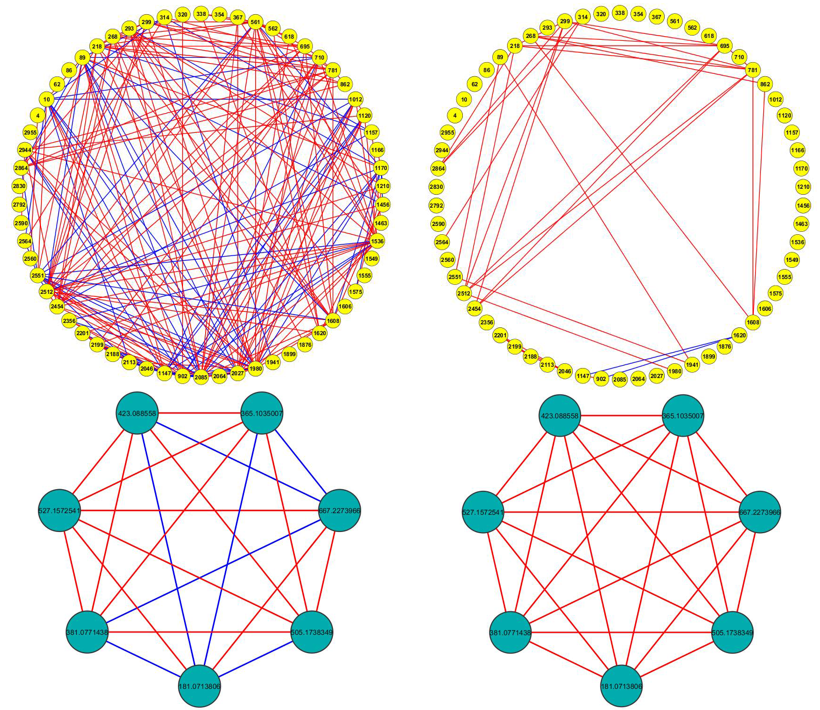

Supplement: Additional file 5: — Changes in co-expression between metabolite pairs in specific network modules in female fly metabolome. Top panel: Within module changes in co-expression in the red module across two conditions of larval temperature, 18°C (left) and 27°C (right). Edges are colored as in Figure 3. Both location, and relative order of each node in the module are the same across the two temperature conditions. Bottom panel: Changes in correlation among a subset of annotated metabolites from the magenta module. Mass to charge values (mz values) are shown inside the nodes. Annotated mz’s are 423.0885 (adenosyl- homocysteine), 365.1036 (lactose/maltose/melibiose/sucrose), 527.1572 (maltotriose), 381.0771 (lactose/maltose/melibiose/sucrose), 181.0713 (glucose/galactose), 505.1738 (maltotriose), and 667.2271 (maltotetraose). [file 12918_2014_139_MOESM5_ESM.tiff]
