# Supplementary material for: Invariance and plasticity in the Drosophila melanogaster metabolomic network in response to temperature
Source: BMC Syst Biol. 2014 Dec 24;8:139. doi: 10.1186/s12918-014-0139-6 (PMC4302152; doi:10.1186/s12918-014-0139-6)
Supplement: Additional file 6: — Metabolic pathways (b), enriched in differentially co-expressed modules (a) in male Drosophila . These modules were identified by the DiffCoEx algorithm [22], as significantly altered in response to developmental temperature exposures. The number of metabolites in the input list that overlapped (b) with the reference list of all metabolites after quality control (c), along with the identification of these metabolites (d) is shown. [file 12918_2014_139_MOESM6_ESM.docx]

| **Module**  **(a)** | **Enriched pathways**  **(b)** | **Overlap size (c)** | **Pathway size (d)** |
| --- | --- | --- | --- |
| Black | - Betaxanthin biosynthesis via dopamine | 3 | 5 |
| Green | - Glycogen degradation - Lactose/melibiose degradation - Trehalose biosynthesis and degradation | 5  4  4 | 9  4  4 |
| GreenYellow | - N-acetlyglucosamine degradation | 2 | 3 |
| Purple | - CDP- diaclyglycerol biosynthesis/ Glycerol-3-phosphate shuttle | 2 | 3 |
| Turquoise | - Purine deoxyribonucleoside degradation - Lipoate biosynthesis - Lactose/melibiose degradation | 4  3  2 | 9  5  3 |
| Yellow | - Glycolysis - Lactose/melibiose degradation | 4  2 | 11  3 |

Metabolic pathways enriched in differentially co-expressed modules in response to developmental temperature exposure in males.

**Additional file 5**. Metabolic pathways (b), enriched in differentially co-expressed modules (a) in male *Drosophila*. These modules were identified by the DiffCoEx algorithm (22), as significantly altered in response to developmental temperature exposures. The number of metabolites in the input list that overlapped (b) with the reference list of all metabolites after quality control (c), along with the identity of these metabolites (d) is shown. Only pathways with an adjusted p-value < 0.01 were selected as enriched. We used *Mummichog* (32) to populate this table. *Mummichog* flag parameters that were changed from default were, -n set to “fly”, and -u set to “FTMS”.
