# Supplementary material for: Invariance and plasticity in the Drosophila melanogaster metabolomic network in response to temperature
Source: BMC Syst Biol. 2014 Dec 24;8:139. doi: 10.1186/s12918-014-0139-6 (PMC4302152; doi:10.1186/s12918-014-0139-6)
Supplement: Additional file 7: — Metabolic pathways (b), enriched in differentially co-expressed modules (a) in female Drosophila. These modules were identified by the DiffCoEx algorithm [22], as significantly altered in response to developmental temperature exposures. The number of metabolites in the input list that overlapped (b) with the reference list of all metabolites after quality control (c), along with the id of these metabolites (d) is shown. [file 12918_2014_139_MOESM7_ESM.docx]

Metabolic pathways enriched in differentially co-expressed modules in response to developmental temperature exposure in female.

| **Module**  **(a)** | **Enriched pathways**  **(b)** | **Overlap size (c)** | **Pathway**  **size (d)** |
| --- | --- | --- | --- |
| Blue | - N-acetlyglucosamine degradation | 2 | 3 |
| Brown | - Purine deoxyribonucleosides degradation - Salvage pathways of adenine, hypoxanthine, and their nucleosides - Degradation of purine ribonucleosides | 4  4  3 | 8  15  10 |
| Turquoise | - Salvage pathways of adenine, hypoxanthine, and their nucleosides - Purine deoxyribonucleosides degradation - NAD biosynthesis | 4  3  3 | 15  8  8 |

**Additional file 6**. Metabolic pathways (b) enriched in differentially co-expressed modules (a) in female Drosophila. These modules were identified by the DiffCoEx algorithm (22), as significantly altered in response to developmental temperature exposures. The number of metabolites in the input list that overlapped (b) with the reference list of all metabolites after quality control (c), along with the identity of these metabolites (d) is shown. Only pathways with an adjusted p-value < 0.01 were selected as enriched. We used *Mummichog* (32) to populate this table. *Mummichog* flag parameters that were changed from default were, -n set to “fly”, and -u set to “FTMS”.
