# Supplementary material for: Invariance and plasticity in the Drosophila melanogaster metabolomic network in response to temperature
Source: BMC Syst Biol. 2014 Dec 24;8:139. doi: 10.1186/s12918-014-0139-6 (PMC4302152; doi:10.1186/s12918-014-0139-6)
Supplement: Additional file 8: — Mass-to-charge ratios of metabolites in the enriched pathways detected by mummichog and status of their identity confirmation using the METLIN database. [file 12918_2014_139_MOESM8_ESM.docx]

Mass-to-charge ratios of metabolites in the enriched pathways detected by *Mummichog* and status of their identity confirmation using the METLIN database

| **Metabolite identified by *Mummichog* (a)** | **Adduct (b)** | **m/z (c)** | **METLIN Match (d )** |
| --- | --- | --- | --- |
| ADENOSINE 3',5'-BISPHOSPHATE (PAP) | [M+H]^+^ | 428.0343138 | YES |
| 3-5-ADP |  | 429.0390035 | NO |
| 3,4-DIHYDROXYPHENYLACETATE (CPD-782) | [M+2H]^2+^ | 85.02783791 | YES |
| ADENOSYL-HOMO-CYS | [M+K]^+^ | 423.0885264 | YES |
| TREHALOSE- 6 PHOSPHATE | [M+H]^+^ | 423.088558 | YES |
| TREHALOSE | [M+Na]^+^ | 365.1035007 | YES |
| ADENOSINE MONOPHOSPHATE (AMP) | [M+H]^+^ | 348.0698628 | YES |
| XANTHINE | [M+H]^+^ | 153.0400782 | YES |
| ADENINE | [M+H]^+^ | 136.0615224 | YES |
| GLUCOSE 1-PHOSPHATE (GLC-1-P) | [M+H]^+^ | 261.0364146 | YES |
| MALTOTRIOSE | [M+Na]^+^ | 527.1572541 | YES |
| MALTOSE/SUCROSE/LACTOSE | [M+Na]^+^ | 365.1035007 | YES |
| VALINE | [M+K]^+^ | 156.041601 | YES |
| PROLINE |  | 117.0733274 | NO |
| PHENYLALANINE | [M+H]^+^ | 166.0853471 | YES |
| INOSINE | [M+Na]^+^ | 291.0686469 | YES |
| DEOXYADENOSINE |  | 254.1026471 | NO |
| DEOXYINOSINE | [M+H+Na]^2+^ | 138.0421118 | YES |
| HYPOXANTHINE | [M+H]^+^ | 137.045126 | YES |
| 4Α-CARBOXY-5Α-CHOLESTA-8,24-DIEN-3Β-OL (CPD-4702) | [M+K]^+^ | 467.2941076 | YES |
| 4Α-HYDROXYMETHYL-4Β-METHYL-5Α-CHOLESTA-8,24-DIEN-3Β-OL (CPD-4575) | [M+K]^+^ | 226.1793293 | YES |
| 44-DIMETHYL-CHOLESTA-812-24-TRIENOL | [M+Na]^+^ | 495.3259472 | YES |
| SPHINGOSINE 1-PHOSPHATE (CPD3DJ-11366) | [M+H]^+^ | 448.2406349 | YES |
| SPERMINE | [M+Na]^+^ | 225.2038101 | YES |
| S-ADENOSYLMETHIONINAMINE |  | 378.1469402 | NO |
| LACTOSE | [M+Na]^+^ | 381.0771438 | YES |

**Additional file 1**. Metabolites (a) in enriched pathways in Drosophila identified by *Mummichog* (Li et al 2013) in this study. The observed m/z value of each metabolite (c), its adduct (b), and the status of its confirmation in the METLIN database (46).
