# Supplementary material for: Invariance and plasticity in the Drosophila melanogaster metabolomic network in response to temperature
Source: BMC Syst Biol. 2014 Dec 24;8:139. doi: 10.1186/s12918-014-0139-6 (PMC4302152; doi:10.1186/s12918-014-0139-6)
Supplement: Additional file 9: — Details of the DIFFCOEX algorithm used to identify both differentially co-expressed and significantly preserved modules in this study. [file 12918_2014_139_MOESM9_ESM.docx]

**Details of the DIFFCOEX algorithm used to identify both differentially co-expressed and significantly preserved modules in this study**

**Detecting differentially co-expressed modules in male and female *D. melanogaster***. A soft-thresholding parameter value (β1) of 5 was used. The cutreeHybrid function was invoked, with cutHeight = 0.995, and minClustersize = 30, to extract modules from the hierarchical tree.

**Detecting significantly preserved modules in male and female *D. melanogaster*.** A soft-thresholding parameter value (β1) of 5 was used. The cutreeHybrid function was invoked, with cutHeight = 0.992, and minClustersize = 30, to extract modules from the hierarchical tree.
